# Autotaxin and LPA Receptors Represent Potential Molecular Targets for the Radiosensitization of Murine Glioma through Effects on Tumor Vasculature

**DOI:** 10.1371/journal.pone.0022182

**Published:** 2011-07-20

**Authors:** Stephen M. Schleicher, Dinesh K. Thotala, Amanda G. Linkous, Rong Hu, Kathleen M. Leahy, Eugenia M. Yazlovitskaya, Dennis E. Hallahan

**Affiliations:** 1 School of Medicine, Vanderbilt University, Nashville, Tennessee, United States of America; 2 Division of Nephrology, Department of Medicine, Vanderbilt-Ingram Cancer Center, Vanderbilt University, Nashville, Tennessee, United States of America; 3 Neuro-Oncology Branch, National Cancer Institute, National Institutes of Health, Bethesda, Maryland, United States of America; 4 Department of Radiation Oncology, Washington University School of Medicine, St. Louis, Missouri, United States of America; 5 Siteman Cancer Center, Washington University School of Medicine, St. Louis, Missouri, United States of America; 6 Mallinckrodt Institute of Radiology, Washington University School of Medicine, St. Louis, Missouri, United States of America; Bauer Research Foundation, United States of America

## Abstract

Despite wide margins and high dose irradiation, unresectable malignant glioma (MG) is less responsive to radiation and is uniformly fatal. We previously found that cytosolic phospholipase A2 (cPLA_2_) is a molecular target for radiosensitizing cancer through the vascular endothelium. Autotaxin (ATX) and lysophosphatidic acid (LPA) receptors are downstream from cPLA_2_ and highly expressed in MG. Using the ATX and LPA receptor inhibitor, α-bromomethylene phosphonate LPA (BrP-LPA), we studied ATX and LPA receptors as potential molecular targets for the radiosensitization of tumor vasculature in MG. Treatment of Human Umbilical Endothelial cells (HUVEC) and mouse brain microvascular cells bEND.3 with 5 µmol/L BrP-LPA and 3 Gy irradiation showed decreased clonogenic survival, tubule formation, and migration. Exogenous addition of LPA showed radioprotection that was abrogated in the presence of BrP-LPA. In co-culture experiments using bEND.3 and mouse GL-261 glioma cells, treatment with BrP-LPA reduced Akt phosphorylation in both irradiated cell lines and decreased survival and migration of irradiated GL-261 cells. Using siRNA to knock down LPA receptors LPA1, LPA2 or LPA3 in HUVEC, we demonstrated that knockdown of LPA2 but neither LPA1 nor LPA3 led to increased viability and proliferation. However, knockdown of LPA1 and LPA3 but not LPA2 resulted in complete abrogation of tubule formation implying that LPA1 and LPA3 on endothelial cells are likely targets of BrP-LPA radiosensitizing effect. Using heterotopic tumor models of GL-261, mice treated with BrP-LPA and irradiation showed a tumor growth delay of 6.8 days compared to mice treated with irradiation alone indicating that inhibition of ATX and LPA receptors may significantly improve malignant glioma response to radiation therapy. These findings identify ATX and LPA receptors as molecular targets for the development of radiosensitizers for MG.

## Introduction

Malignant glioma (MG) is characterized by neovascularization and invasion into surrounding brain parenchyma, which negatively impacts successful resection [1]. Unresectable MG is uniformly fatal with a median survival time of one year [2]. Therefore, response to chemotherapeutic and radiation approaches are essential to control growth and spread. However, disease progression occurs within the field of irradiation despite increased dosage. The identification of new molecular targets for drug development could significantly improve therapeutic outcomes in MG.

ATX was originally discovered as a tumor motility protein [3] from melanoma cells and is a type II membrane protein secreted by cells [4], [5]. It is known to contribute to invasive properties in non-small cell lung cancer [6], renal cell cancer [7] and most recently glioblastoma multiforme (GBM) [8]. ATX converts extracellular lysophosphatidylcholine (LPC) to lysophosphatidic acid (LPA) through its lysophospholipase D activity [9]. It has been demonstrated that specific G-protein coupled receptors (GPCRs) mediate the cellular effects of LPA, such as proliferation and migration in cancer [10]. Three of seven identified LPA-specific receptors, Edg-2/LPA1, Edg-4/LPA2, and Edg-7/LPA3, belong to the endothelial cell differentiation gene (EDG) family and share approximately 60% homology [11], [12]. ATX and LPA receptors are both highly expressed in MG, and invading MG cells show increased gene expression of ATX compared to cells in the originating tumor core [8], [13]. Octa-decenyl thiophosphate (OTP), a LPA receptor agonist showed radioprotective effect in cell lines transfected with LPA2 but not LPA1 and LPA3 [14]. OTP also protected intestinal crypt cell viability in irradiated wild type but not in irradiated LPA2 null mice.[14] Recently, VPC-12249, an LPA1 and LPA3 receptor antagonist was reported to attenuate radiation-induced pneumonitis in mice [15]. LPA receptors on endothelial cells can contribute to angiogenesis through the increased expression and production of neovascularizing factors such as interleukin (IL) -6, IL-8 and vascular endothelial growth factors (VEGF) [16]. The ability of a tumor to recruit and generate new vasculature leads to growth and invasion into surrounding tissue.

The protein kinase B (PKB) or Akt pathway has been implicated in various diseases like cancer, diabetes and autoimmunity [17], [18]. Increased activation of Akt have been reported in melanoma, breast, colon, ovarian, pancreatic and prostrate cancers [18], [19], [20], [21] and implicated as a leading cause of chemo- and radio resistance [21], [22]. The effectiveness of radiotherapy is often limited by the response of the tumor microvasculature [23]. We previously found that ionizing radiation (IR) induces the activation of cytosolic phospholipase A2 (cPLA_2_) in tumor endothelium which leads to the production of LPC and Akt phosphorylation resulting in radioresistance of endothelial cells [24]. Moreover, the inhibition of cPLA_2_ prior to irradiation leads to the disrupted endothelial cell function and the destruction of tumor blood vessels, which translates into suppressed tumor growth [25]. Since LPC is a known substrate for ATX, we hypothesized that ATX and LPA receptors might be the effectors of cPLA_2_ -induced radioresistance in vascular endothelial cells (Fig. S1). Therefore, in this study, we investigated whether ATX and LPA receptors could serve as novel molecular targets for the radiosensitization of MG through enhanced cytotoxic effects in the tumor vasculature by using a dual ATX and LPA receptor inhibitor, α-bromomethylene phosphonate LPA(BrP-LPA)[26], [27] .

We found that ATX and LPA receptor inhibition enhanced radiation-induced endothelial cell death, disrupted endothelial cell biological function, and reduced glioma cell viability and migration. Most importantly, BrP-LPA treatment prior to irradiation repressed glioma tumor growth *in vivo*. These findings suggest that ATX and LPA receptors represent potential molecular targets to enhance the efficacy of radiation therapy.

## Results

### Inhibition of ATX and LPA receptors enhances cell death in irradiated HUVEC and bEnd.3 vascular endothelial cells

**Figure 1:**
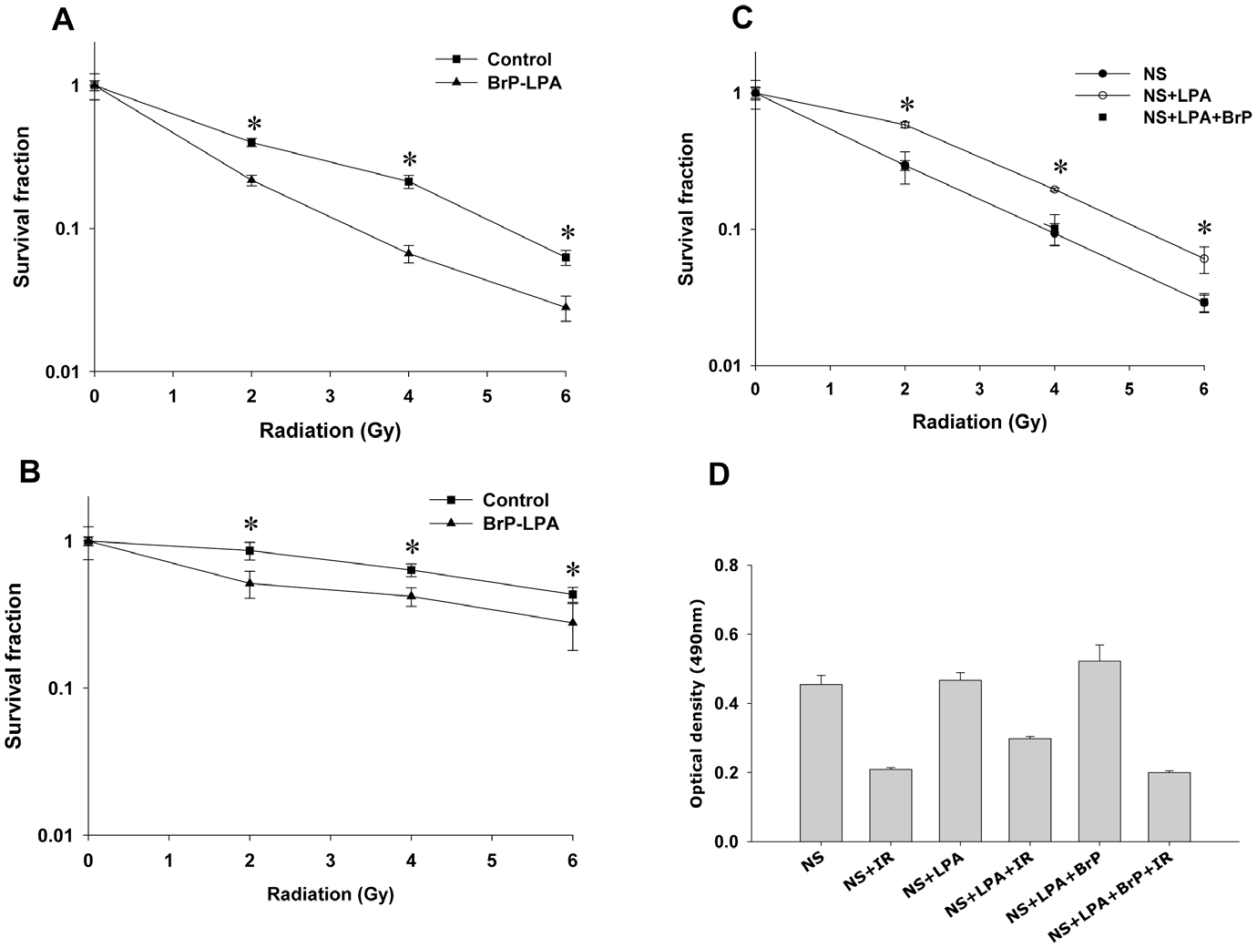
Inhibition of ATX and LPA receptors enhances cell death in irradiated vascular endothelial cells. (A) HUVEC or (B) bEnd.3 cells were treated with vehicle control (▪) or 5 µM BrP-LPA (▴) for 45 min prior to irradiation. (C) HUVEC were treated with 0.1% fatty acid free BSA (•), 10 µM LPA (○) or 10 µM LPA plus 5 µM BrP-LPA (▪) in serum free medium for 45 min prior to irradiation. The cells were then irradiated with 0, 2, 4 and 6 Gy and plated for clonogenic survival assay. After 2–3 wks, cells were stained with 1% methylene blue and colonies consisting of >50 cells were counted by microscopy. Surviving colonies were normalized for plating efficiency. Shown are average survival fractions and SEM from three experiments; * p<0.05. (D) Equal numbers of HUVEC were plated in 96 well plates and treated with carrier control 3% fatty acid free BSA, 10 µM LPA or 10 µM LPA with 5 µM BrP-LPA in serum free medium for 45 min prior to irradiation. After 96 h, the cell viability was determined using a colorimetric cell proliferation assay (Promega). Shown is the average absorbance at 490 nm with SEM from three experiments; * p<0.05.

We recently found that cPLA_2_ inhibition enhanced cell death in irradiated 3B11 murine vascular endothelial cells [25]. We hypothesized that LPC produced by radiation-activated cPLA_2_ is converted to LPA by ATX and could activate signaling pathways through its LPA receptors. To determine whether inhibition of ATX and LPA receptors leads to radiosensitization of brain microvascular endothelial cells, we used α-bromomethylene phosphonate LPA (BrP-LPA), an inhibitor of ATX activity and a pan-antagonist of four LPA receptors [26], [27], to assess clonogenic survival of irradiated HUVEC. Treatment with BrP-LPA enhanced cell death in irradiated cells at 2, 4, and 6 Gy (Fig. 1A). At 2 Gy, ATX and LPA receptors inhibition led to a 45% decrease in survival compared to cells treated with vehicle control (surviving fractions 0.23 versus 0.07, respectively, p = 0.009; Fig. 1A). Similar effects were observed in bEnd.3 cells (Fig. 1B). The most pronounced effect occurred at 4 Gy, in which treatment with BrP-LPA enhanced radiation-induced cell death by 70% compared to corresponding control cells (survival fractions 0.51 versus 0.92, respectively, p = 0.04; Fig. 1B).

To determine whether the LPA receptors mediate radiation-induced cellular responses, we treated HUVEC with 10 µM LPA in serum free medium with or without 5 µM BrP-LPA. HUVEC treated with exogenous LPA showed increased survival and radioprotection compared to untreated cells (Fig. 1C and 1D). Treatment with BrP-LPA abrogated this radioprotective effect triggered by exogenous LPA suggesting that radiosensitization by BrP-LPA could be mediated through the ATX and LPA receptors.

**Figure 2:**
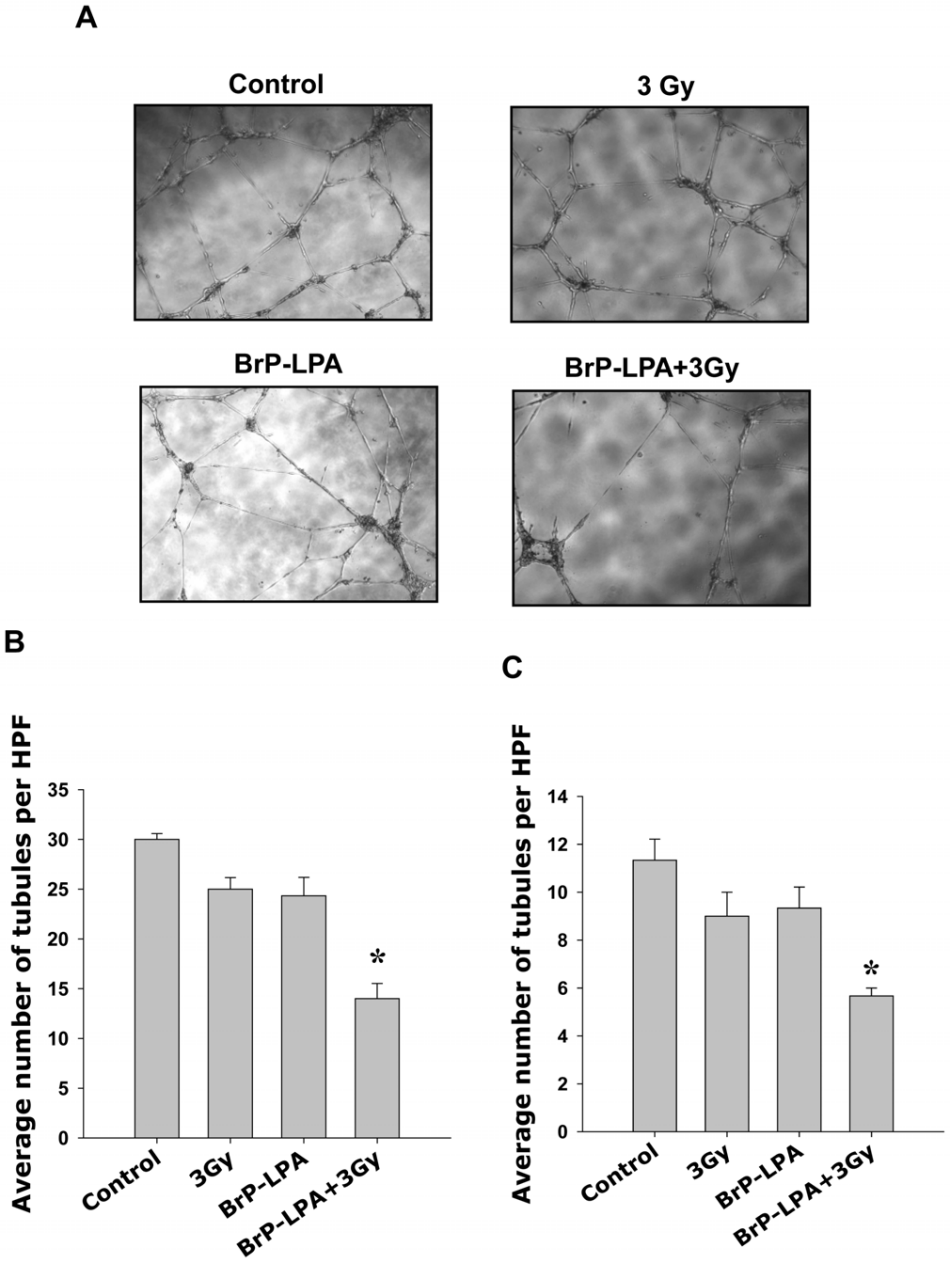
Inhibition of ATX and LPA receptors attenuates tubule formation in irradiated vascular endothelial cells. (A, B) HUVEC or (C) bEnd.3 cells were plated on matrigel-coated 96 well plates and treated with vehicle control or 5 µM BrP-LPA for 45 min prior to irradiation with 3 Gy. (A) Shown are the representative photomicrographs of tubule formation taken 16 h after irradiation. (B) Tubule formation was quantified as number of tubules per high power field (HPF). Shown are bar graphs of mean number of tubules per HPF relative to control and SEM from three experiments; * p<0.05.

ATX and LPA receptors inhibition attenuates tubule formation in irradiated vascular endothelial cells. To determine if ATX and LPA receptors inhibition disrupts the ability of endothelial cells to form capillary-like tubule structures, HUVEC were plated on Matrigel-coated 96-well plates. Cells were treated with 5 µM BrP-LPA or H_2_0 as vehicle control for 45 min before irradiation (3 Gy). Tubule formation was monitored and recorded at 16 h after irradiation. Treatment with 3 Gy or BrP-LPA alone resulted in a slight decrease in the ability of cells to form tubule structures (Fig. 2A and B). However, the combination of ATX and LPA receptor inhibition with irradiation resulted in a 53% attenuation of tubule formation compared to corresponding controls (Fig. 2B, p = 0.0006). Similar results were observed in bEnd.3 cells (Fig. 2C) in which ATX and LPA receptors inhibition produced a 50% reduction in the number of tubules formed compared to its corresponding control (p = 0.0039).

### Inhibition of ATX and LPA receptors attenuates migration in irradiated endothelial cells

**Figure 3:**
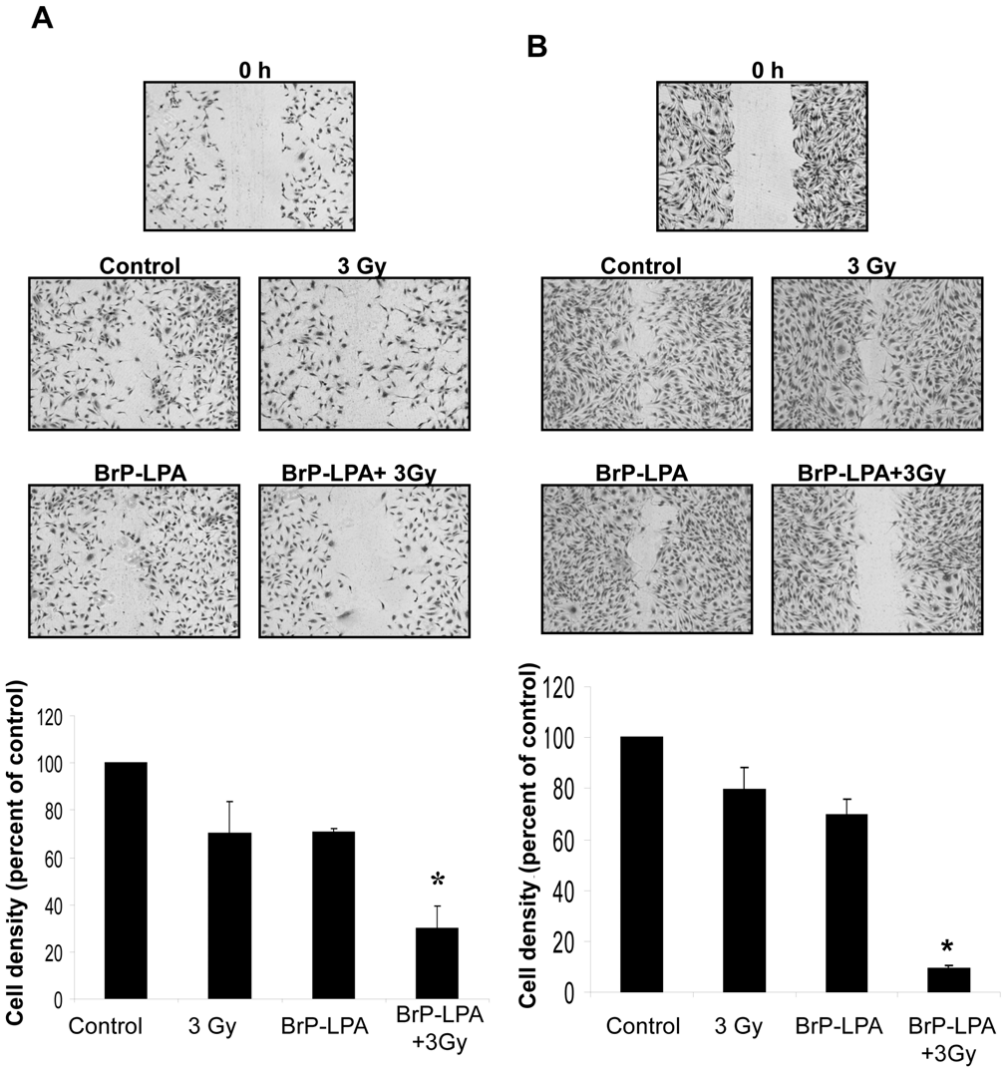
Inhibition of ATX and LPA receptors reduces irradiated vascular endothelial cell migration. (A) bEnd.3 or (B) HUVEC were plated on 60 mm plates and allowed to grow to 70% confluency. The semi-confluent cell layer was scraped using a sterile pipette tip to create a scratch devoid of cells. The remaining cells were treated with vehicle control or 5 µM BrP-LPA for 45 min prior to irradiation with 3 Gy. Migration was observed at 36 h. Cells were fixed with ethanol and stained with 1% methylene blue. Migrated cells were counted and normalized to surrounding cell density per HPF. Shown are representative photomicrographs and bar graphs representing the mean percentages of migrating cells relative to corresponding controls with SEM from three experiments, * p<0.05.

To determine whether inhibition ATX and LPA receptors results in reduced endothelial cell migration, a scratch assay was performed by treating HUVEC and bEnd3 cells with 5 µM BrP-LPA or H_2_0 as vehicle control for 45 min prior to irradiation (3 Gy). Migrated cells ware counted and normalized relative to surrounding cell density at 24 h after irradiation. Combined treatment with BrP-LPA and 3 Gy significantly attenuated endothelial cell migration compared to radiation alone in both HUVEC (Fig. 3A, 70% versus 30%) and bEnd3 cells (Fig. 3B, 80% versus 9%). These results indicate that BrP-LPA is able to attenuate migration in both HUVEC and bEnd3 cells.

### Inhibition of ATX and LPA receptors attenuates migration and enhances cell death in irradiated GL261 glioma cells

**Figure 4:**
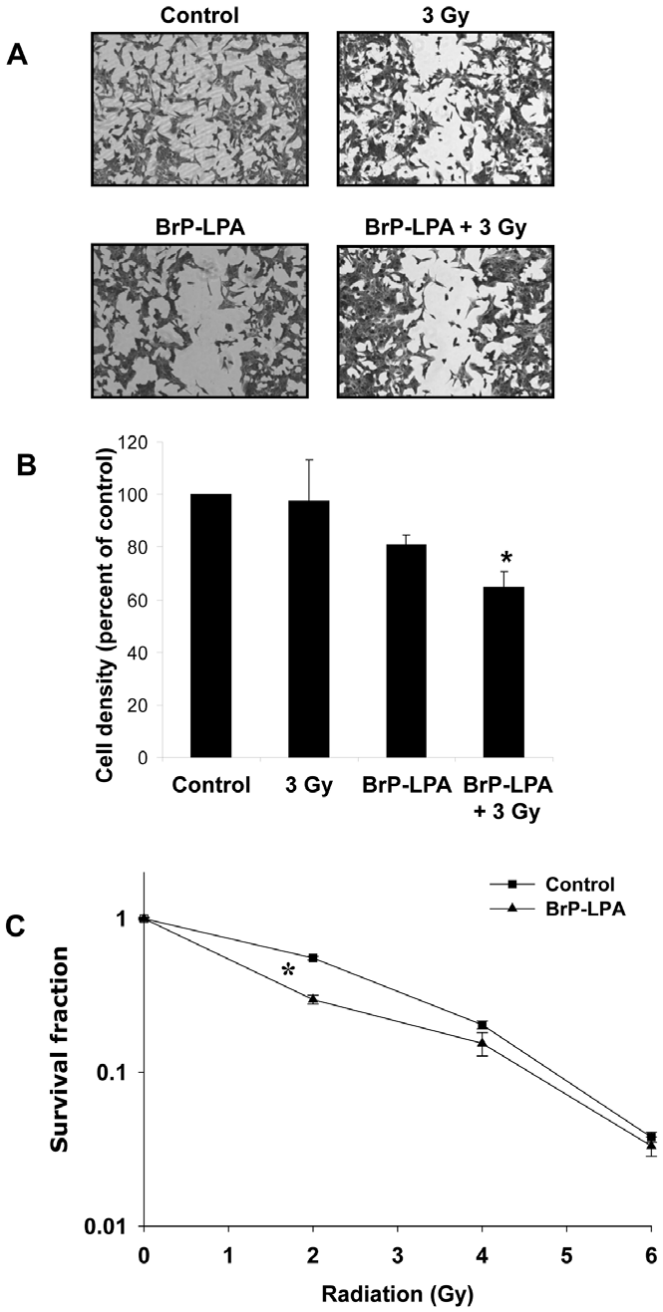
Inhibition of ATX and LPA receptors attenuates migration and enhances cell death in irradiated GL261 cells. (A, B) Mouse glioma GL261 cells were plated on 60 mm plates and allowed to grow to 70% confluency. Plates were scraped with a pipette tip to create a scratch devoid of cells and treated with vehicle control or 5 µM BrP-LPA for 45 min before irradiation with 3 Gy. After 24 h, cells were fixed and stained with methylene blue. Migrated cells were counted and normalized to surrounding cell density per HPF. Shown are representative photomicrographs (A) and a bar graph (B) representing the mean percentages of migrating cells relative to corresponding controls with SEM from three experiments; * p<0.05. (C) For clonogenic survival assay, GL261 cells were plated and allowed to attach. After 6 h, cells were treated vehicle control or with 5 µM BrP-LPA for 45 min and irradiated with 0, 2, 4, and 6 Gy. After 10 days, surviving colonies (>50 cells) were counted and normalized for plating efficiency. Shown is the clonogenic survival curve and mean surviving fractions and SEM from three experiments; * p<0.05.

Since glioma cells express high levels of ATX and multiple LPA receptors [8] , we investigated the effects of BrP-LPA on cell migration and colony formation in GL261 cells. Treatment of GL261 cells with either 5 µM BrP-LPA or 3 Gy alone resulted in a minor reduction in cell migration (81% and 98% of control, respectively; Fig. 4A and 4B). However, combined treatment with BrP-LPA and irradiation reduced GL261 migration to 65% of control (Fig. 4B, p = 0.018). In clonogenic survival studies, irradiated GL261 cells treated with 5 µM BrP-LPA showed a modest but significantly reduced survival at the radiation dose of 2 Gy compared to cells treated with radiation alone (Fig. 4C).

### Inhibition of ATX and LPA receptors disrupts pro-survival signals in irradiated bEnd.3 and GL261 cells grown in co-culture

**Figure 5:**
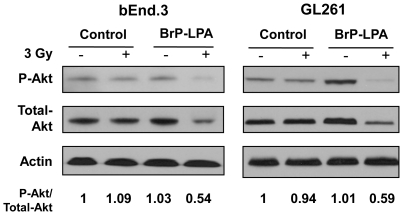
Inhibition of ATX and LPA receptors reduces Akt phosphorylation in irradiated glioma and endothelial cells grown in co-culture. bEnd.3 and GL261 cells were grown in co-culture for 24 h. Cells were treated with vehicle or 5 µM BrP-LPA control for 45 min before treatment with 3 Gy. Cells were lysed at 5 min after irradiation. Shown are immunoblot analyses using specific antibodies to phospho-Akt^Thr308/Ser473^, total Akt, and actin. Numbers represent the ratios of phospho-Akt to total Akt protein normalized to actin (fold change relative to control).

We previously found that irradiation induces cPLA_2_ activation in endothelial cells, which leads to LPC production and the subsequent activation of Akt in tumor endothelium [24]. ATX expressed by glioma cells may contribute to this pro-survival signaling by converting LPC to LPA, thereby activating LPA receptors. To investigate whether ATX and LPA receptors inhibition can disrupt Akt activation, bEnd.3 and GL261 cells were grown in co-culture and treated with 5 µM BrP-LPA or vehicle control 45 min prior to 3 Gy irradiation. Treatment with 5 µM BrP-LPA prior to 3 Gy irradiation reduced Akt activation in bEnd.3 endothelial cells relative to treatment with 3 Gy radiation alone (0.54 versus 1.09 fold change relative to control, respectively; Fig. 5). In GL261, Akt phosphorylation was also reduced in cells treated with 5 µM BrP-LPA prior to 3 Gy irradiation compared to treatment with either drug or irradiation alone (0.59 versus 1.01 versus 0.94 fold change relative to control, respectively; Fig. 5).

**Figure 6:**
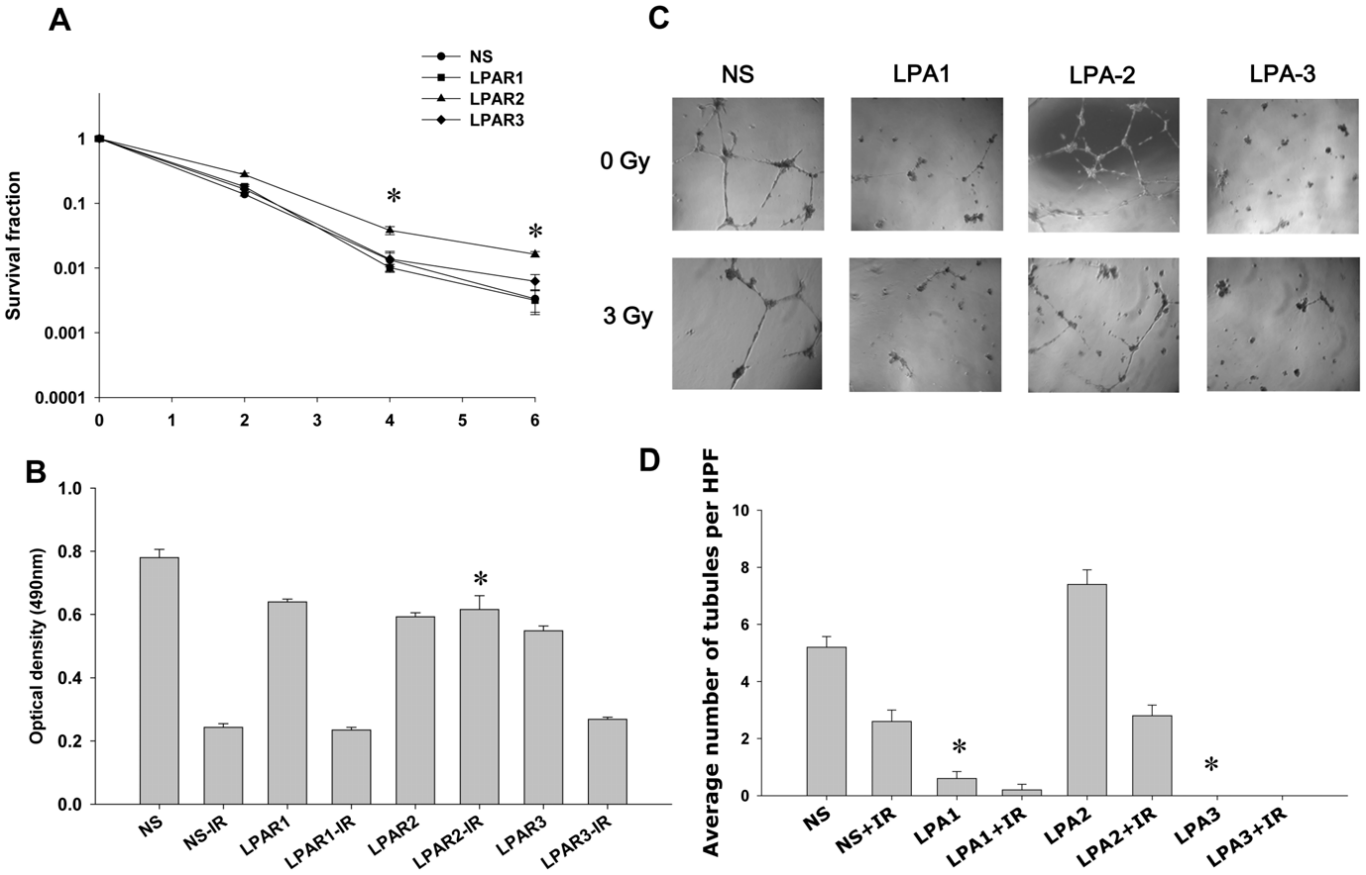
Effects of knockdown of LPA1, LPA2 and LPA3 on survival and tubule formation of irradiated HUVEC. HUVEC were transiently transfected with the non-silencing (•) or LPA1- (▪), LPA2- (▴) or LPA3- (♦) specific siRNA. (A) The knocked down cells were then irradiated with 0, 2, 4 and 6 Gy and plated for clonogenic survival assay. After 2–3 wks, cells were stained with 1% methylene blue and colonies consisting of >50 cells were counted by microscopy. Surviving colonies were normalized for plating efficiency. Shown are average survival fractions and SE from three experiments; * p<0.05. (B) Equal numbers of knocked down cells were plated in 96 well plates and irradiated with 3 Gy. After 96 h, the cell viability was determined using a colorimetric cell proliferation assay (Promega). Shown is the average absorbance at 490 nm with SEM from three experiments; * p<0.05. (C, D) The knocked down cells were plated on matrigel-coated 96 well plates and treated with 3 Gy. (C) Shown are the representative photomicrographs of tubule formation taken 16 h after treatment. (D) Tubule formation was quantified as number of tubules per HPF. Shown are bar graphs of mean tubules per HPF relative to control and SEM from three experiments; * p<0.05.

Knockdown of LPA1 and LPA3 receptors leads to decreased tubule formation in HUVEC. To determine the role of LPA receptors in clonogenic survival, proliferation and tubule formation of irradiated vascular endothelial cells, we used siRNA to knock down LPA1, LPA2 or LPA3 in HUVEC. Knockdown of either LPA1 or LPA3 had no effect on cell survival in irradiated HUVEC compared to the nonsilencing control (Fig. 6A and 6B). In contrast, knockdown of LPA2 showed increased survival of irradiated HUVEC in both the clonogenic and proliferation assays (Fig. 6A and 6B). In tubulogenesis assays, compared to nonsilencing control, knockdown of LPA1or LPA3 demonstrated significant decrease in tubule formation (Fig. 6C and 6D) whereas knockdown of LPA2 showed increased tubule formation (Fig. 6C and 6D). Upon treatment with 3 Gy, tubules were undetectable in HUVEC with knocked down LPA1or LPA3; while knockdown of LPA2 did not show any change in tubule formation compared to nonsilencing control. These results indicate that LPA2 is involved in radiation-induced cell death and decreased tubule formation in endothelial cells, and likely, does not take part in radiosensitizing effects of BrP-LPA. On the contrary, while LPA1 and LPA3 do not play noticeable role in radiation-dependent viability of endothelial cells, they are critical for tubule formation.

### Inhibition of ATX and LPA receptors represses tumor growth in irradiated GL261 mouse model

**Figure 7:**
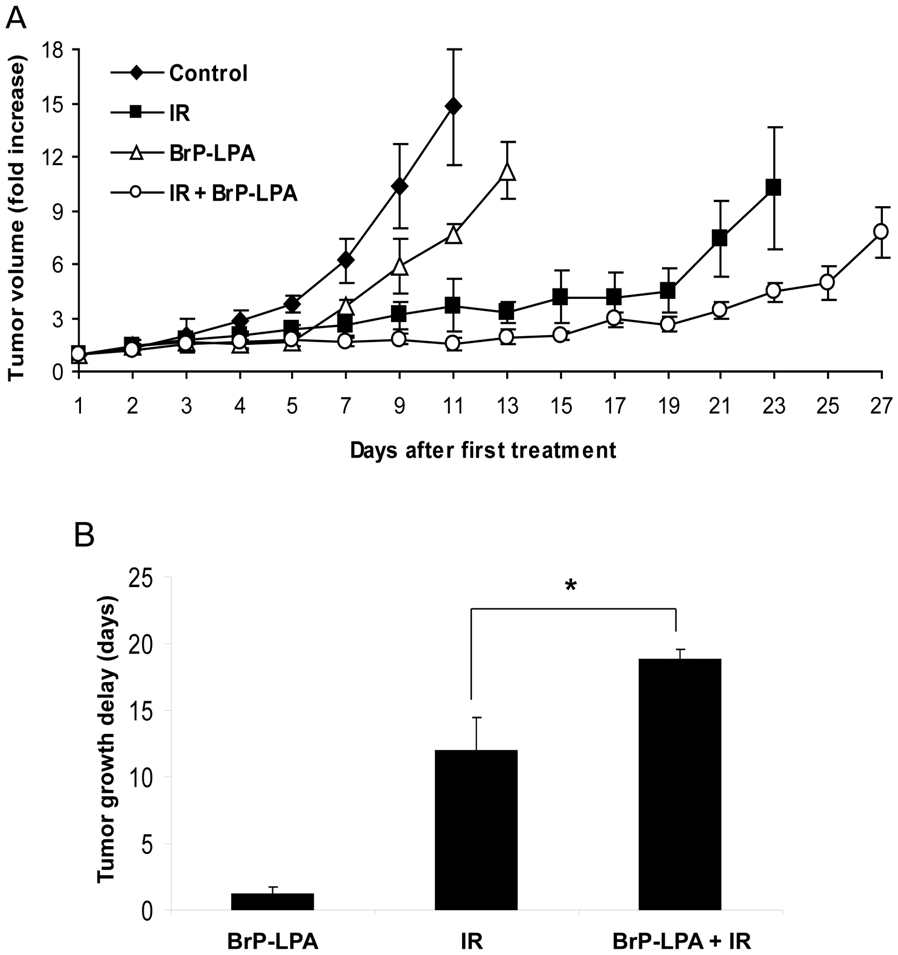
Inhibition of ATX and LPA receptors represses tumor growth in irradiated GL261 mouse model. GL261 cells were injected into the hind limbs of nude mice. Tumors were irradiated with 3 Gy for 5 consecutive days for a total of 15 Gy. Mice were treated with 3 mg/kg BrP-LPA or vehicle control for 45 min prior to irradiation on days 1, 3, and 5. (A) Shown are mean tumor volumes with SEM from each treatment group of 5 mice. (B) Tumor growth delay was calculated as the number of days for tumors to reach a 6-fold volume increase compared to control. Shown is a bar graph representing the mean tumor growth delay with SEM from each treatment group of 5 mice; * p<0.05.

To determine the efficacy of ATX and LPA receptor inhibition *in vivo*, a heterotopic mouse brain tumor model was used. GL261 cells (1×10^5^) were injected s.c. into the right hind limbs of nude mice. Tumor-bearing mice were then treated with vehicle alone, irradiation alone (5 fractions of 3 Gy), 3 mg/kg BrP-LPA alone, or 3 mg/kg BrP-LPA for 45 min followed by irradiation (5 fractions of 3 Gy). Treatment with either BrP-LPA or irradiation alone delayed tumor growth relative to control (1.2 days and 12.0 days, respectively; Fig. 7). The most pronounced tumor growth delay, however, was observed in mice receiving a combination of BrP-LPA and irradiation. In this treatment group, the tumor growth delay was 6.8 days relative to treatment with irradiation alone (p = 0.03). This difference remained statistically significant (p = 0.0005) after using Holm's correction for multiple comparisons, and a 2^2^ factorial design study of tumor growth delay revealed an additive interaction between BrP-LPA and irradiation.

## Discussion

Despite the use of multiple treatment modalities including chemotherapy and radiation therapy, unresectable MG is characterized by poor cure rates and high frequencies of recurrence. A significant contributor to poor treatment outcomes is the highly motile phenotype exhibited by MG that allows invasion into surrounding parenchyma and makes it incurable by localized therapies [28]. Tumor growth and spread require a vascular framework that depends on endothelial cell migration and angiogenesis [29]. In addition, the endothelium is important because it has been shown that tumor vascular responses to ionizing radiation largely affect tumor radiosensitivity [23], [24]. While it is possible that some cytotoxicity could occur within the normal vasculature, it would be limited since radiation is primarily cytotoxic to rapidly dividing cells. Tumor endothelial cells proliferate 20 to 2000 times faster than normal tissue endothelium[30].

We previously found that cPLA_2_ activation in endothelial cells occurs within minutes of exposure to IR. Inhibition of cPLA_2_ leads to decreased survival and angiogenesis in irradiated endothelial cells, as well as enhanced lung cancer radiosensitivity *in vivo* [25]. The significance of this pathway may be amplified in MG, where radiosensitizing the tumor vasculature may not only improve tumor response to radiotherapy, but may also diminish angiogenesis-dependent tumor growth and spread. In the present study, we found that ATX and LPA receptors, molecular targets downstream of cPLA_2_, can be inhibited in MG to enhance radiation-induced destruction of endothelial cell functions and improve tumor response to irradiation. Using a clonogenic survival assay, we first found that inhibition of ATX and LPA receptors prior to irradiation enhances endothelial cell death. In HUVEC, reduced clonogenicity was observed after treatment with BrP-LPA and radiation compared to treatment with radiation alone. Similar effects were observed in brain microvascular endothelial cells (Fig. 1). These results support previously observed enhanced cytotoxicity after inhibition of the upstream molecular target, cPLA_2_, in HUVEC and 3B11 tumor vascular endothelial cells [25].

LPA is present in the serum in the concentrations of 5–10 µmol/L [31]. Exogenous addition of LPA to serum free medium protected HUVEC from radiation damage (Fig. 1C and 1D) confirming a critical role for LPA in radiosensitivity of vascular endothelial cells. Addition of BrP-LPA to HUVEC abrogated LPA-induced radioprotection (Fig. 1C) suggesting involvement of LPA receptors in activation of pro-survival radioprotective signaling pathway in vascular endothelial cells. Interestingly, the effects of BrP-LPA were not limited to endothelial cells, and enhanced cell death was also observed in irradiated GL261 glioma cells (Fig. 4). One potential mechanism may involve diminished activation of the pro-survival kinase Akt. LPA, which is produced by ATX and acts through LPA receptors, has been shown to transiently phosphorylate Akt in C6 glioma cells [32]. Moreover, cPLA_2_ inhibition reduces phospho-Akt in irradiated endothelial cells [25]. Akt inhibitor IV has been shown to inhibit colony formation in malignant glioma cell line SF763. It has been shown that Akt activation is linked to glioma radio-resistance and down regulation of Akt signaling induces apoptosis and reduced motility of these cells [33]. Consistent with these findings, we demonstrated that ATX and LPA receptors inhibition reduced Akt phosphorylation in both irradiated brain endothelial and glioma cells (Fig. 5).

We also found that ATX and LPA receptors inhibition enhances the efficacy of radiation to disrupt endothelial cell tubule formation and migration. In HUVEC and bEnd.3 endothelial cells, treatment with 5 µM BrP-LPA prior to 3 Gy significantly reduced endothelial cell tubule formation and migration compared to treatment with 5 µM BrP-LPA or irradiation alone (Fig. 2 and Fig. 3). Angiogenesis is a complex process that requires both endothelial cell migration and tubule formation [29]. The growth and spread of cancer requires an underlying vascular framework to provide nutrients for expanding cells [34], [35]. MG is particularly prone to invade normal brain which complicates the ability of current treatment regimens to prevent recurrence [28]. Therefore, the disruption of endothelial cell angiogenic functions like tubule formation and migration may be particularly important in MG. In the present study, the anti-migratory effects of BrP-LPA were not limited to endothelial cells, and ATX and LPA receptors inhibition also reduced the ability of irradiated GL261 glioma cells to migrate in the scratch assay (Fig. 4). Although ATX and LPA receptors have never been studied in combination with radiation, previous studies have suggested important roles for these molecular targets in tubule formation and cell motility. In ovarian cancer cell lines, LPA contributes to angiogenesis by stimulating the neovascularizing factors IL-6, IL-8, and vascular endothelial growth factor (VEGF) expression [16], and ATX modulates cell responsiveness to VEGF [36]. Diminished mRNA levels of LPA receptors are associated with abolished HUVEC migration in response to upstream signaling lipids LPC and LPA [36], and ATX has been shown to induce LPA receptor-dependent glioma cell motility [8]. Interestingly, LPA induces the phosphorylation of the regulatory light chain of myosin II in glioma cells [37], which may play a role in the disrupted motility we observed in irradiated GL261 cells (Fig. 5).

The ATX and LPA receptors inhibitor BrP-LPA we used has also been studied in A549 non-small cell lung cancer [27] and MDA-MB-231 breast cancer [38] cells at doses ranging from 10 to 40 µM, but its effects have never been studied in endothelial cells or in conjunction with radiation. BrP-LPA is both an ATX and pan LPA receptor antagonist [27], making it difficult to study the differential effects of each molecular target individually. We found potential therapeutic benefit of inhibiting both these targets downstream from cPLA_2_, thus indicating that the development of more potent, specific inhibitors of ATX and LPA receptors is needed. A specific ATX substrate LPC is abundantly present in plasma, ranging 100–200 µM [39]. In the presence of such high concentration of a substrate, BrP-LPA is less likely to inhibit ATX activity. It is more likely that BrP-LPA is acting at the level of the LPA receptors. LPA1 and LPA3 have been reported to be involved in inflammation leading to radiation pneumonitis [15]. LPA2 has been reported to protect against radiation induced intestinal injury [14]. In our studies, we observed that LPA2 knockdown produced increased viability and proliferation in irradiated HUVEC compared to nonsilencing controls, while knockdown of LPA1 or LPA3 did not show any effect (Fig. 6A, and B). These results suggest that LPA2, but neither LPA1 or LPA3, is an important factor in transduction of radiation-induced cell death in vascular endothelial cells. Further, we observed that knockdown of either LPA1 or LPA3 led to no tubule formation in HUVEC (Fig. 6 C and D), demonstrating that LPA1 and LPA3 are critical for endothelial cell functions. These results also imply that LPA1 and LPA3, but not LPA2, involved in radiosensitizing effects of BrP-LPA. This observation is supported by the results from another group stating that LPA1 and LPA3 are involved in inflammation leading to radiation pneumonitis and are targets for radiosensitization [15]. Therefore, we conclude that LPA1 and LPA3 on vascular endothelial cells are molecular targets for radiosensitization and tumor control as endothelium plays a vital role in tumor growth and invasion, Based on the published literature, in addition to inhibiting LPA1 and LPA3 on endothelial cells, BrP-LPA could also function by inhibiting ATX and LPA receptors on cancer cells (Fig. S1). This possible mode of BrP-LPA action requires further investigation.

Summarizing the present study, we characterize the important role of ATX and LPA receptors in the vascular response to irradiation. We found that the inhibition of these targets enhances the ability of radiation to induce endothelial cell death, disrupt endothelial cell tubule formation, and attenuate endothelial cell migration. Most importantly, inhibition of ATX and LPA receptors along with radiation therapy in a heterotopic mouse model repressed overall GL261 tumor growth (Fig. 7). Moreover, radiosensitization of the tumor vasculature may contribute to tumor growth delay. These findings suggest that pre-treatment with drugs that inhibit ATX and LPA receptors may significantly improve malignant glioma response to radiation therapy. Clinical trials with such drugs will be required before any clinical benefit can be determined.

## Materials and Methods

### Cell cultures and treatment

Mouse brain microvascular (bEnd.3) cells were obtained from ATCC and maintained in DMEM with 10% FBS and 1% penicillin/streptomycin (Life Technologies, Gaithersburg, MD). Human umbilical vein endothelial cells (HUVEC) were obtained from and quality tested by Lonza and maintained in endothelial cell growth medium (Lonza, Switzerland). GL261 cells were obtained from Dr. Yancie Gillespie (University of Alabama-Birmingham, Birmingham, AL) and maintained in DMEM-F12 with 10% FBS and 1% penicillin/streptomycin. GL261 cells were MAP tested by Department of Comparative Medicine at Washington University and were negative for mycoplasma. All cells were grown in a 5% CO_2_ incubator at 37°C. To inhibit ATX and LPA receptors, we used α-bromomethylene phosphonate LPA (BrP-LPA) which was purchased from Echelon (Salt Lake City, UT). For the radiation of cells, a PANTAK pmc1000 x-ray machine with a 0.1 Cu^+^ 2.5 AL filter was used at a dose rate of 88.7 cGy/min.

### Clonogenic survival

Cells were plated in triplicate onto 6 cm plates and allowed to attach for 6 h. Cells were then treated with 5 µM BrP-LPA or H_2_0 as vehicle control for 45 min before irradiation with 0, 2, 4, or 6 Gy. Cells were incubated at 37°C in 5% CO_2_ for 10–20 days depending on cell type. Cells were fixed with 70% ethanol and stained with 1% methylene blue and colonies comprised of more than 50 cells were counted using microscopy. Average survival fraction of the treatment was calculated as (number of colonies/number of cells plated)/(number of colonies of corresponding control/number of cells plated) with standard error.

### Colorimetric cell proliferation assay

Cell proliferation was determined using cell titer 96 Aqueous Non-Radioactive Cell Proliferation Assay reagent (Promega). The assay was done following the manufacturers protocol. Briefly equal numbers of HUVEC cells of various treatments were plated into different wells of a 96-well plate. Cell viability was determined colorimetrically by measuring absorbance at 490 nm after 96 hours after treatment. Experiments were done in triplicate and average fold changes relative to control and standard errors were calculated.

### Matrigel-based tubule formation assay

96-well plates were coated with 75 µL per well of Matrigel (BD Biosciences, San Jose, CA) and incubated overnight at 37°C. bEnd.3 cells (1.5×10^4^ ) or HUVEC cells (10^4^) were plated over the solidified matrigel and allowed to attach at 37°C for 45 min prior to treatment. Cells were treated with 5 µM BrP-LPA or H_2_0 as vehicle control for 45 min and irradiated with 3 Gy. Capillary-like tubule formation was monitored in control wells (16–24 h) using a microscope, photomicrographs of the cells were recorded, and the number of tubules per 4 randomly selected high power fields (HPF) was counted. Tubule formation was quantified as the average number of tubules per HPF normalized to control. The experiment was done in triplicate and the mean and standard error were calculated for each treatment group.

### Cell migration assay

BEnd.3, HUVEC, or GL261 cells were plated in triplicate onto 6 cm plates and allowed to grow to 70% confluency. The semi-confluent cell layer was scratched with a sterile pipette tip to create a scratch devoid of cells and plates were washed twice with PBS to remove nonadherent cells and debris. Cells were treated with 5 µM BrP-LPA or H_2_0 for 45 min prior to irradiation with 3 Gy, and then incubated at 37°C in 5% CO_2_. Control plates were monitored for cell migration (20–24 h). Cells were fixed with 70% ethanol and stained with 1% methylene blue. To quantify migration, cells in 3 randomly selected high power fields (HPF's) in the scratched area were counted and normalized for surrounding cell density. Mean and standard error for each treatment group were calculated.

### Transfection of siRNA

The silencer select pre-designed siRNAs targeting LPA1, LPA2, and LPA3 and nontarget control siRNA were obtained from Applied Biosystems/Ambion. siRNAs were delivered into cells using HiPerFect transfecting reagent according to the manufacturer's protocol (QIAGEN Valencia, CA). siRNA induced gene knockdown was confirmed by immunoblotting for LPA1, LPA2 and LPA3 48 hours after siRNA deliveryLPA treatment LPA was purchased from Avanti Polar Lipids Inc. LPA was applied to the cells complexed with 3% fatty acid free bovine serum albumin (Sigma chemical Co., St Louis, MO). Briefly, HUVECs cells were serum-starved with endothelial cell growth medium (Lonza) for1 h and treated with 10 µM LPA prior to irradiating or treating with BrP-LPA.

Co-culture Western immunoblot analysis bEnd.3 cells (2.5×10^5^) were plated in 6 well plates and after 24 h, GL261 (5×10^5^) cells were plated onto transwell inserts (Corning Inc., Corning, NY). After co-culture for 24 h, cells were treated with 5 µM BrP-LPA or H_2_0 for 45 min prior to treatment with 3 Gy. After 5 min, bEnd.3 and GL261 cells were harvested for protein extraction using M-PER kit (Pierce, Rockford, IL). Protein concentrations were estimated using BCA Reagent (Pierce, Rockford, IL) and 30 µg of each sample was subjected to Western immunoblot analysis using specific antibodies to phospho-Akt and total Akt (both from Cell Signaling Technologies, Danvers, MA). Antibody to actin (sigma) was used to evaluate protein loading in each lane. Immunoblots were developed using the Western Lightning Chemiluminescence Plus detection system (PerkinElmer, Wellesley, MA) according to the manufacturer's protocol. Band densities were quantitated using BioRad Quantity One software.

### Mice, treatment and tumor growth delay

All animal procedures used in this study were approved by the Department of Comparative Medicine (DCM) at Washington University (Animal Studies Committee approval number 20090226), and the housing and handling of animals followed DCM guidelines. GL261 cells (1×10^5^) were injected into the right hind limb of nude mice. Once tumors were palpable (The average tumor size was 96.82 mm^3^), mice were stratified into four treatment groups of 5 mice representing similar distributions of tumor sizes. Tumors from two groups of mice were irradiated with 3 Gy fractions daily for 5 consecutive days for a total of 15 Gy. These mice received 3 mg/kg BrP-LPA or H_2_0 as vehicle control i.p. 45 min prior to irradiation on the first, third, and fifth day of treatment. The two groups of non-irradiated mice received 3 mg/kg BrP-LPA or H_2_0 alone at the same times as the irradiated mice. Tumor volumes were measured using external caliper. Tumor volumes for each animal were normalized to the initial tumor volume at the start of treatment, and the mean tumor fold increase and standard error were calculated for each treatment group.

### Statistical analyses

The mean and SE of each treatment group were calculated for all experiments. The number of samples is indicated in the description of each figure. Statistical analysis was done using a student's t-test to compare two means with p<0.05 representing statistical significance. For the *in vivo* tumor growth delay study, a 2^2^ factorial design was used. The t-test using Holm's correction for multiple comparisons was used to test significance between group means. The standard deviation used for the multiple comparisons was obtained by pooling the standard deviations of the test groups. The factorial design was also analyzed in terms of variable effects in order to determine if BrP-LPA/IR interaction was synergistic or additive.

## Supporting Information

Figure S1 **Schematic representation of proposed autotaxin (Lyso PLD) signaling in cancer.** Ionizing radiation induces production of lipid second messenger LPC. LPC is converted to LPA by ATX which is highly expressed in cancer cells. LPA in turn activates LPA receptor signaling leading to angiogenesis, cancer cell migration and vascular permeability.
(TIF)
