# Supplementary figures and images for: Autotaxin and LPA Receptors Represent Potential Molecular Targets for the Radiosensitization of Murine Glioma through Effects on Tumor Vasculature
Source: PLoS One. 2011 Jul 20;6(7):e22182. doi: 10.1371/journal.pone.0022182 (PMC3140496; doi:10.1371/journal.pone.0022182)

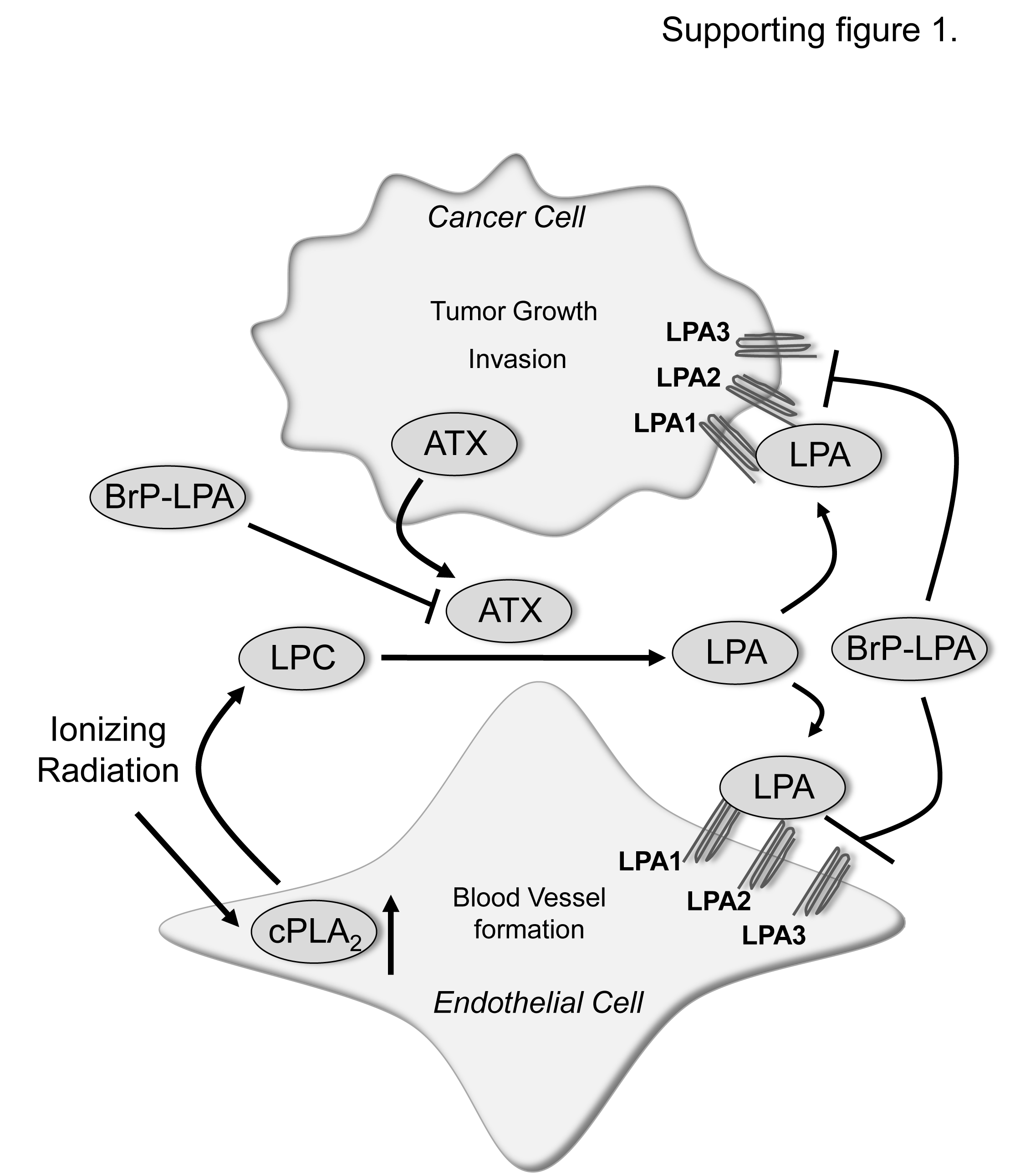

Supplement: Figure S1 — Schematic representation of proposed autotaxin (Lyso PLD) signaling in cancer. Ionizing radiation induces production of lipid second messenger LPC. LPC is converted to LPA by ATX which is highly expressed in cancer cells. LPA in turn activates LPA receptor signaling leading to angiogenesis, cancer cell migration and vascular permeability. (TIF) [file pone.0022182.s001.tif]
